# Non-specific symptoms as a prodrome of immune-related adverse events in patients with non-small cell lung cancer receiving nivolumab: a consecutive analysis of 200 patients

**DOI:** 10.1007/s00432-022-04205-9

**Published:** 2022-07-28

**Authors:** Ryoko Inaba Higashiyama, Hidehito Horinouchi, Aya Kuchiba, Yuji Matsumoto, Shuji Murakami, Yasushi Goto, Shintaro Kanda, Yutaka Fujiwara, Noboru Yamamoto, Yuichiro Ohe

**Affiliations:** 1 Department of Thoracic Oncology, National Cancer Center Hospital, 5-1-1 Tsukiji, Chuo-ku, Tokyo, 104-0045 Japan; 2 Course of Advanced Clinical Research of Cancer, Juntendo University Graduate School of Medicine, Tokyo, Japan; 3 Graduate School of Health Innovation, Kanagawa University of Human Services, Kanagawa, Japan; 4 Biostatistics Division, Center for Research Administration and Support, National Cancer Center, Tokyo, Japan; 5 Department of Thoracic Oncology, Kanagawa Cancer Center, Kanagawa, Japan; 6 Department of Hematology and Medical Oncology, Shinshu University School of Medicine, Nagano, Japan; 7 Department of Thoracic Oncology, Aichi Cancer Center, Aichi, Japan

**Keywords:** Non-small cell lung cancer, Immune checkpoint inhibitors, Immune-related adverse events, Prodrome, Signal symptom

## Abstract

**Purpose:**

Immune checkpoint blockade therapy is the standard treatment for metastatic or refractory non-small cell lung cancer (NSCLC). However, it is associated with immune-related adverse events (irAEs). irAEs are sometimes fatal; however, an efficient method for early irAEs detection has not been developed because their onset timing varies. We examined the significance of non-specific symptoms as a prodrome of irAEs in patients with NSCLC.

**Methods:**

We reviewed consecutive patients who received nivolumab at a dosage of 3 mg/kg every 2 weeks for metastatic NSCLC between December 2015 and August 2017. Patient demographics, irAEs and signal symptoms were recorded. Non-specific symptoms (fever and fatigue) occurred 7 days or earlier before the onset of irAEs were considered signal symptoms. For statistical analyses, the association between irAEs and clinical information, including signal symptoms, was evaluated using Fisher’s exact test and logistic regression.

**Results:**

A total of 200 patients received nivolumab; 131 (65.5%) were male, their median age was 63 years (range 30–83), 174 (87.0%) had performance status of 0–1. Signal symptoms occurred in 38 (19.0%) of the 77 patients (38.5%) who experienced irAEs, and were positively associated with the occurrence of irAEs (*P* < 0.01). Multivariate analysis showed that the occurrence of irAEs was significantly higher in patients with PS 0–1 [odds ratio (OR) 7.01; 95% confidence intervals (CI), 1.69–29.13] and patients experienced signal symptoms (OR 17.30; 95% CI, 6.51–45.99).

**Conclusion:**

The occurrence of signal symptoms could be used in the early detection and management of irAEs in patients during immune checkpoint blockade therapy.

**Supplementary Information:**

The online version contains supplementary material available at 10.1007/s00432-022-04205-9.

## Introduction

Lung cancer remains the leading cause of cancer death globally. Immune checkpoint inhibitors, including nivolumab, have been established as a standard treatment for metastatic or refractory non-small cell lung cancer (NSCLC). Adverse events associated with immune checkpoint inhibitors, especially immune-related adverse events (irAEs), are associated with fatal outcomes and severe decline in organ function and quality of life (Borghaei et al. 2015; Brahmer et al. 2015; Herbst et al. 2016; Rittmeyer et al. 2017). Adequate diagnosis and management of irAEs are the major issues in identifying the appropriate immune checkpoint inhibitor to be used. Several guidelines and reviews regarding irAEs have been published (Brahmer et al. 2018; Davies and Duffield 2017). However, an efficient method for the early detection of irAEs has not been developed, mainly because of different onset timings of irAEs and a wide range of organ systems that can be affected. Moreover, there is little information in the existing guidelines about the prediction and early diagnosis of irAEs. Therefore, we investigated the potential of non-specific prodromal symptoms, such as fever and fatigue, for the early detection of irAEs. Our findings could have clinical significance in the early detection and treatment of NSCLC.

## Materials and methods

We conducted a retrospective analysis of adult patients (aged ≥ 18 years) who were cytologically or histologically diagnosed with advanced/metastatic (i.e., stage IIIc/IV) NSCLC and received nivolumab at a dosage of 3 mg/kg every 2 weeks at the National Cancer Center Hospital (NCCH), Japan, between December 2015 to August 2017; we observed the patients until August 2018 or death. The study was performed as per the NCCH institutional review board-approved protocol (No. 2015-355). All patients with malignant thoracic tumors diagnosed and treated at the Department of Thoracic Oncology, National Cancer Center Hospital who consented to the analysis were included in the analysis, and patients who did not consent to the analysis were excluded.

We reviewed the medical records of these patients and collected the following data regarding their pretreatment characteristics: demographics, efficacy and safety of nivolumab, information about irAEs, and signal symptoms. The patients were followed up every 1–2 weeks at the outpatient clinic. Signal symptoms were defined as non-specific symptoms (fever and fatigue) that were not present at the time of nivolumab initiation and were present at least 7 days prior to the diagnosis of irAEs.

The questionnaire is predetermined and standardized for all patients receiving immune checkpoint inhibitors at our institution. The questionnaires were used by medical staffs, especially nurses, independent of the physician, prior to physician's visit, prior to each administration of nivolumab. The questionnaire consisted of 25 questions that could be answered with a yes or no. The questionnaire was based on the following criteria items. Items are as follows: cough, shortness of breath, dyspnea, itching, rash, skin color change, loss of appetite, diarrhea, bloating, abdominal pain, nausea, joint pain, muscle pain, palpitation, hyperhidrosis, headache, depression, edema, general malaise, jaundice of the conjunctiva bulbar, dry mouth, urine output increase, fever, and blurred vision. Regular systemic work-ups including chest X-ray, chest and abdominal computer tomography (CT), and brain CT or magnetic resonance imaging (MRI) were performed almost regularly every 3 months in the outpatient clinic.

The diagnosis of irAEs was first made by the physician in charge and recorded in the medical records. In this study, two specialists independently reviewed the medical records to confirm whether or not these were irAEs. The CTCAE version 4.0 was used to report adverse events.

The Fisher exact test was used to evaluate the association between the presence or absence of irAEs and the presence or absence of signal symptoms. The multivariate logistic regression model was used to estimate odds ratios (ORs), and 95% confidence intervals (CIs) were used to estimate the association between irAEs and factors related to patient characteristics. The following potential factors were investigated in the logistic regression model: age, sex, Eastern Cooperative Oncology Group (ECOG) performance status (0–1 vs 2–4), smoking history (past or current vs. never), tumor histology (non-squamous vs. squamous), driver gene alteration (positive vs. negative or unknown), year of treatment initiation (2015–2016 vs 2017 or later), and signal symptoms (presence vs. absence). *P *value < 0.05 was considered to indicate statistical significance. All analyses were performed using STATA ver. 12.0 (Stata, College Station, TX, USA).

## Results

**Fig. 1:**
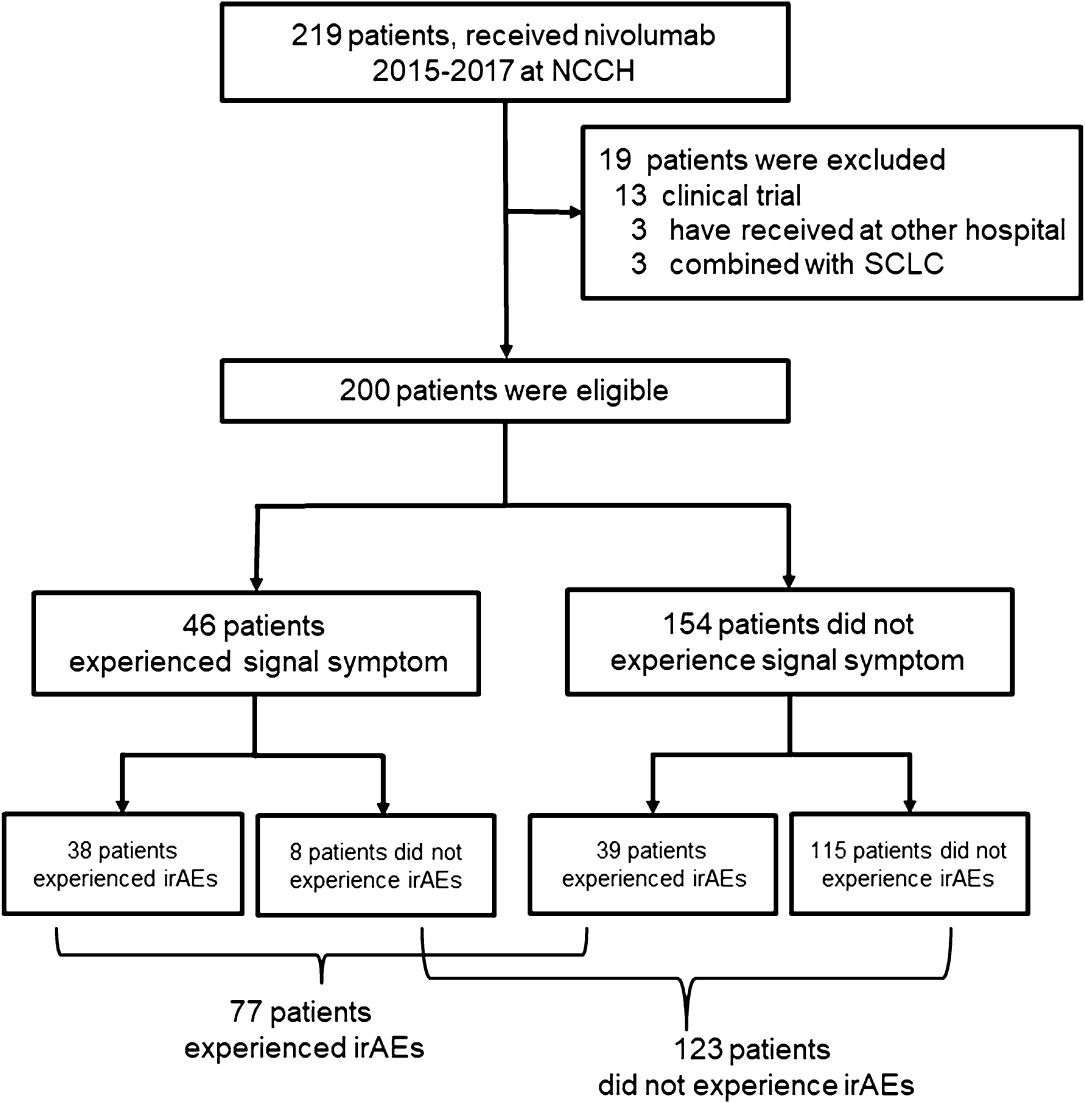
Study flow diagram (*N* = 200)

Two-hundred patients with NSCLC received nivolumab at NCCH, Japan, between December 2015 and August 2017 (Fig. 1). The baseline patient characteristics are shown in Supplemental Table 1. All patients received at least one complete cycle of nivolumab, the median age of the patients was 63 years (range 30–83 years), 131 patients (65.5%) were male, 26 (13%) had a PS of 2 or more, 144 (72%) were past or current smokers, and 40 (20%) had driver gene alterations, mainly epidermal growth factor receptor (EGFR) mutations. Forty-nine (24.5%) patients had lung squamous NSCLC, 151 (75.5%) had non-squamous NSCLC, 130 (65%) had adenocarcinoma, 6 (3%) had large cell neuro-endocrine carcinoma (LCNEC), 3 (1.3%) had pleomorphic carcinoma, and 12 (6%) had others. The median observation period from the start of administration of nivolumab was 275 days (minimum 12, maximum 1017).

IrAEs appeared in 77 patients (38.5%). In patients with irAEs, the median time from the start of administration of nivolumab to the onset of irAEs was 29 days (0–518 days). There were 20 cases of Grade 1, 29 cases of Grade 2, 22 cases of Grade 3, 4 cases of Grade 4 and 2 of grade 5 irAEs. Thyroid dysfunction, being the most common irAE, was found in 19 cases (9.5%), followed by 16 cases (8%) of dermatitis, 15 (7.5%) of pneumonitis, 12 (7%) of colitis, 4 of adrenal dysfunction (2%), 4 of liver dysfunction (2%), 1 of acute renal dysfunction 1 cases (0.5%), 1 of autoimmune myocardial damage (0.5%), 1 of autoimmune cystitis (0.5%), 1 of autoimmune pancreatitis (0.5%), and 1 of uveitis (0.5%). The most common irAE of grade 3 or higher was pneumonitis, which occurred in 11 patients (Supplemental Table 2).

Signal symptoms appeared in 46 of the 200 patients treated with nivolumab: fever and fatigue were observed in 21 (10.5%) and 31 (15.5%) cases, respectively, and simultaneous occurrence of fever and fatigue was observed in 6 cases. In patients who presented with signal symptoms, the median duration from the start of nivolumab administration to the onset of signal symptoms was 29 days (range, 0–518 days), and the median duration from the appearance of signal symptoms to the onset of irAEs was 28 days (range 7–259 days).

Signal symptoms were observed in 38 cases in the group with irAEs and 8 cases in the group without irAEs (Table 1). Among the patients who experienced signal symptoms, 82.6% had irAEs, and among those who did not, 25.3% had irAEs (OR 14.0, 95% CI 6.10–32.04).

**Table 1:** Relationship between signal symptoms and immune-related adverse events (irAEs)

		irAEs		
		Yes	No	Total
Signal symptoms	Yes	38	8	46
	No	39	115	154
	Total	77	123	200

irAEs; immune-related adverse events

**Fig. 2:**
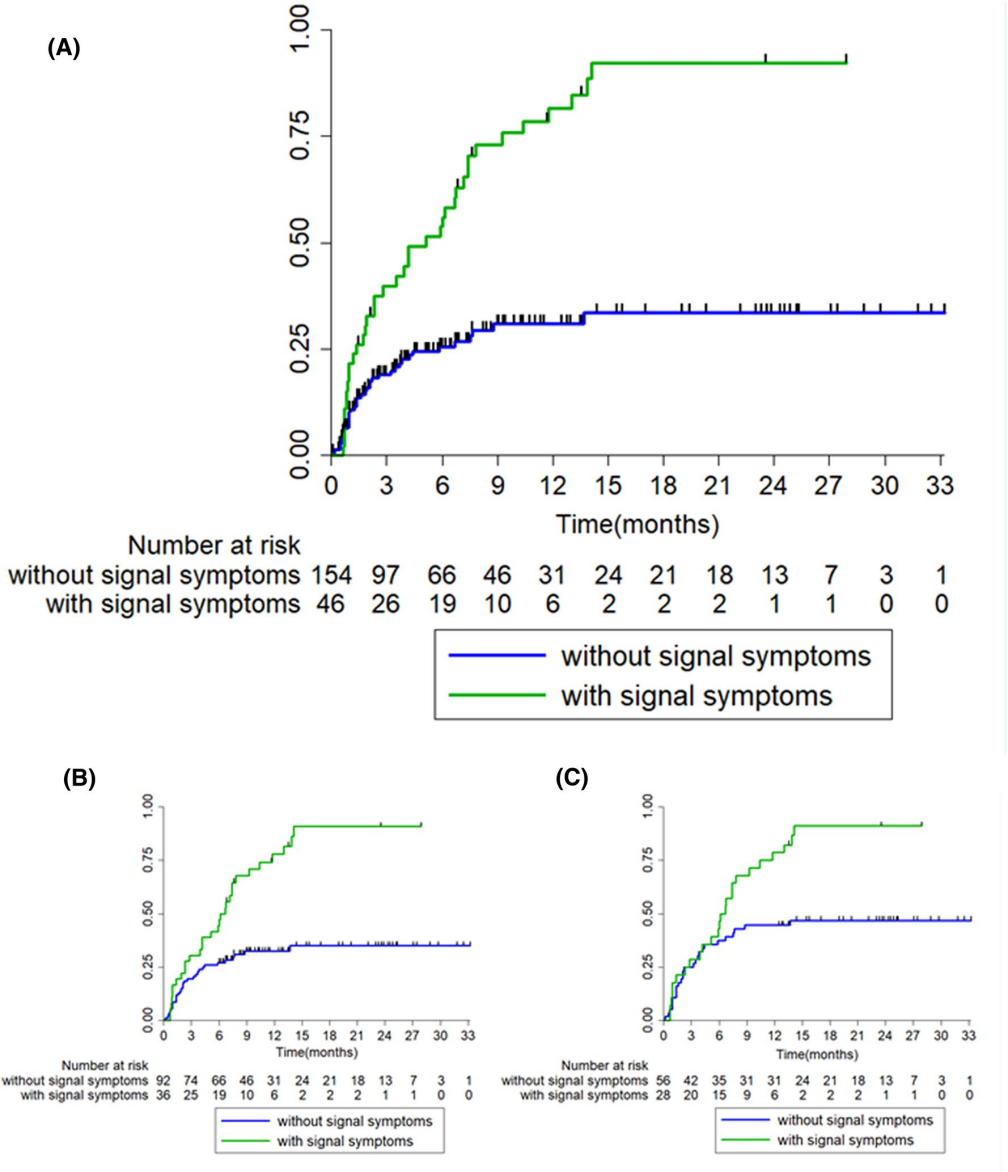
Immune-related adverse events (irAEs) estimate Kaplan–Meier curves for irAEs estimate in **A** all patients, **B** excluded patients who died within a year and **C** excluded patients who died within 6 months

To minimize the impact of cancer-related death as a competing risk, we examined the association between the presence or absence of signal symptoms and irAEs in 84 patients who were alive at least 1 year after the start of nivolumab administration (Fig. 2). We analyzed any correspondence between signal symptoms and irAEs but found no specific association (Table 2). A similar trend was confirmed in analyses conducted with different follow-up periods to minimize competing risks (Fig. 2, Supplemental Tables 2 and 3).

**Table 2:** Frequency of immune-related adverse events (irAEs) per signal symptoms

		irAEs		Endocrine disorder		Diarrhea, colitis		Pneumonitis		Dermatitis		Liver dysfunction	
		+	−	+	−	+	−	+	−	+	−	+	−
Signal symptom	+	38	8	14 (1)	8	1 (1)	8	7 (3)	8	6 (2)	8	1 (2)	8
	−	39	115	9	115	10 (1)	115	6 (1)	115	8 (2)	115	1 (2)	115
	ORs	14.00		22.36		1.44		16.77		10.78		14.38	
		6.10–32.04		7.57–66.28		0.22–10.04		4.74–59.97		3.14–37.57		1.38–120.95	
Fever	+	12	3	4 (1)	3	0	3	3 (2)	3	1 (2)	3	0 (2)	3
	−	39	115	9	115	10	115	6	115	8	115	1	115
	ORs	11.80		17.04		−		19.18		4.79		−	
		3.16–43.99		3.29–88.14		−		3.61–104.39		0.63–39.03		−	
Fatigue	+	20	5	7	5	1 (1)	5	4 (1)	5	5	5	1	5
	−	39	115	9	115	10	115	6	115	8	115	1	115
	ORs	11.80		17.89		2.30		15.33		14.38		23.00	
		4.15–33.54		4.72–67.85		0.24–21.651		3.26–72.24		3.43–60.18		1.25–423.41	

*irAEs* immune-related adverse events, *ORs* odds ratios. Cases in parentheses are those with overlapping irAEs and are not included in the calculation of the odds ratio

Multivariable logistic regression analysis showed that PS and signal symptoms were significantly associated with the incidence of irAEs (OR for PS 7.01, 95% CI 1.69–29.13; OR for signal symptom 17.30, 95% CI 6.51–45.99) (Table 3). CRP values before nivolumab treatment were not significantly associated with the subsequent development of irAEs.

**Table 3:** Multivariate analysis of immune-related adverse events (irAEs) outcomes (*N* = 200)

irAEs	*N*	ORs	*p*	95% CI	
Age					
≧70	46	1			
< 70	154	1.15	0.76	0.470	2.824
Sex					
Female	69	1			
Male	131	2.45	0.06	0.978	6.120
**ECOG PS**					
2–3	26	1			
0–1	174	7.01	0.01	1.689	29.129
Smoking history					
Never smoker	56	1			
Smoker (past or current)	144	2.74	0.05	0.986	7.611
Tumor histology					
Sq	49	1			
Non-sq	151	1.46	0.39	0.616	3.475
Driver gene alternation					
Positive (EGFR or other)	40	1			
Negative	160	2.93	0.07	0.923	9.328
Year of treatment initiation					
2017–	25	1			
2015–2016	175	1.57	0.44	0.504	4.912
Treatment line of nivolumab					
3 or more	112	1			
2	88	1.50	0.32	0.674	3.331
**Signal symptom**					
No	154	1			
Yes	46	17.30	< 0.01	6.510	45.988

*irAEs* immune-related adverse events, *ORs* odds ratios, *95% CI* 95% confidence intervals, *ECOG PS* Eastern Cooperative Oncology Group performance status, *Sq* squamous cell carcinoma

## Discussion

This is the first study to report that non-specific signal symptoms can help in the early detection of irAEs. We observed that signal symptoms occurred at a median duration of 25.5 days before the onset of irAEs. It was confirmed that the frequency of subsequent irAEs was significantly higher in patients who experienced signal symptoms than in those who did not (82.6% vs. 25.3%). Early detection of irAEs could be achieved by focusing on non-specific signal symptoms during the induction phase of immune checkpoint inhibitors.

In this study, a few non-specific symptoms were screened using a predetermined questionnaire. The odds ratio for subsequent irAEs in patients with signal symptoms was 14.0 (95% CI 6.10–32.04). It is reported that 58–69% of patients experience the occurrence of some type of irAEs with the administration of immune checkpoint inhibitors (Borghaei et al. 2015; Brahmer et al. 2015; Herbst et al. 2016; Rittmeyer et al. 2017). Lawson et al. (2022) reported that poor PS is associated with the development of irAE, but this association was not confirmed in our study. Multiple guidelines for irAE management have been published, including guidelines by the American Society of Clinical Oncology and by the National Comprehensive Cancer Network. These guidelines focus on the administration of immunosuppressive agents and hormone replacement therapy (Brahmer et al. 2018). They recommend prompt treatment intervention for irAEs to avoid discontinuation of immune checkpoint inhibitors (Ryder et al. 2014; Davies and Duffield 2017; El Majzoub et al. 2019). Furthermore, these guidelines emphasize the importance of early detection of irAEs. Based on the current analysis, it might be possible to select patients at high risk for irAEs by identifying signal symptoms in advance (median time of 28 days).

The development of signal symptoms may be related to several cytokines that are produced in association with irAEs. Valpione et al. (2018) reported a positive correlation between low blood interleukin (IL)-6 levels before treatment and ipilimumab-induced irAEs. Another study reported significantly elevated blood IL-6 levels in a population of patients who experienced irAEs with the administration of anti-PD-1 antibody (Tanaka et al. 2020). Cytokines such as IL-6 that are produced via the same mechanism as irAEs, may cause symptoms such as fever, fatigue, and headache before the onset of irAEs. Recently, early increase in inflammatory cytokine levels in the presence of a PD-L1 checkpoint inhibitor was shown to be predictive of the efficacy of the inhibitor in patients with NSCLC (Ozawa et al. 2019). Our study is consistent with this report when combined with studies showing that early-onset irAEs are associated with the efficacy of nivolumab in NSCLC (Teraoka et al. 2017; Haratani et al. 2018).

Nevertheless, this study has several limitations. First, owing to the retrospective nature of the study, there could be a possibility of bias in the data analyzed for signal symptoms and irAEs. However, we believe we have minimized recall bias by analyzing information from a predetermined questionnaire that was answered by all patients who received immune checkpoint inhibitors before outpatient visits. In addition, the questionnaire was completed mainly by nurses, independent of the physician. Second, the observation period varied from patient to patient. Patients who have been administered immune checkpoint inhibitors for a longer period may be more likely to develop irAEs. To determine the effect of observation period on the occurrence of irAEs, we compared the incidence of irAEs in both patients with relatively long and short observation periods. The timing of nivolumab initiation was not associated with irAE development in the logistic regression analysis. Although the patient population was analyzed according to whether the patients were observed for more than 1 year from the start of nivolumab administration, signal symptoms were more frequent in patients who developed irAEs regardless of the length of the observation period.

## Conclusions

Signal symptoms (fever and fatigue) are associated with subsequent irAE episodes in patients undergoing nivolumab treatment. By focusing on signal symptoms, preparation for irAEs in advance could be made possible in the clinical setting.

## Supplementary Information

Below is the link to the electronic supplementary material.

Supplementary file1 (DOCX 22 KB)

## Data Availability

The datasets generated during the current study are not publicly available due to ethical restrictions, but are available from the corresponding author on reasonable request.
