# Supplementary material for: Non-specific symptoms as a prodrome of immune-related adverse events in patients with non-small cell lung cancer receiving nivolumab: a consecutive analysis of 200 patients
Source: J Cancer Res Clin Oncol. 2022 Jul 28;149(7):3185–91. doi: 10.1007/s00432-022-04205-9 (PMC10314872; doi:10.1007/s00432-022-04205-9)
Supplement: Supplementary file 1 — Supplementary file1 (DOCX 22 KB) [file 432_2022_4205_MOESM1_ESM.docx]

Supplemental Table1. Patients demographics and clinical characteristics (N=200)

|  | N=200 | % or range |
| --- | --- | --- |
| Age (median) | 63 | 30-83 |
| Sex  female / male | 69/131 | 34.5/65.5 |
| ECOG PS  0-1 / 2-3 | 174/26 | 87/13 |
| Smoking status  Smoker (past or current) / Never smoker | 144/56 | 72/28 |
| Tumor histology |  |  |
| Non-sq / Sq | 151/49 | 75.5/24.5 |
| Driver gene alternation |  |  |
| Positive (EGFR or other) / Negative or unknown | 40/160 | 20/80 |
| Treatment line of nivolumab |  |  |
| 2 / 3 or more | 88/112 | 44/56 |
| Cycles of dose of nivolumab when irAEs developed (median) | 5 | 1-30 |
| Signal symptoms (SSs) | 46 | 23 |
| fever | 21 | 10.5 |
| fatigue | 31 | 15.5 |
| Days after 1^st^ nivolumab when SSs appeared | 29 | 0-518 |
| Year of treatment initiation |  |  |
| 2015-2016 / 2017- | 175/25 | 87.5/12.5 |

ECOG PS; Eastern Cooperative Oncology Group performance status, Sq; squamous cell carcinoma, EGFR; epidermal growth factor receptor

Supplemental Table2. Summary of immune-related adverse events (irAEs) (N=72)

| irAEs | Any grade | Grade1 | Grade2 | Grade3 | Grade4 | Grade5 |
| --- | --- | --- | --- | --- | --- | --- |
| Any irAEs | 77 | 20 | 29 | 22 | 4 | 2 |
| Thyroid dysfunction | 19 | 5 | 13 | 0 | 1 | 0 |
| Dermatitis | 16 | 10 | 6 | 0 | 0 | 0 |
| Pneumonitis | 15 | 1 | 3 | 9 | 0 | 2 |
| Diarrhea, Colitis | 12 | 3 | 3 | 5 | 1 | 0 |
| Adrenal dysfunction | 4 | 1 | 1 | 2 | 0 | 0 |
| Liver dysfunction | 4 | 0 | 2 | 1 | 1 | 0 |
| Acute kidney injury | 1 | 0 | 0 | 1 | 0 | 0 |
| Other | 6 | 0 | 1 | 4 | 1 | 0 |

irAEs; immune-related adverse events.

Supplemental Table3. Frequency of immune-related adverse events (irAEs) per signal symptom among the patients who were alive at least 1 year from the beginning of nivolumab (N=128).

|  | | irAEs | | Endocrine disorder | | Diarrhea, Colitis | | Pneumonitis | | Dermatitis | | Liver dysfunction | |
| --- | --- | --- | --- | --- | --- | --- | --- | --- | --- | --- | --- | --- | --- |
|  |  | ＋ | － | ＋ | － | ＋ | － | ＋ | － | ＋ | － | ＋ | － |
| Signal symptom | ＋ | 30 | 6 | 13(1) | 6 | 1(1) | 6 | 4(2) | 6 | 6(2) | 6 | 1(2) | 6 |
|  | － | 30 | 62 | 6 | 62 | 8(1) | 62 | 3(1) | 62 | 8(2) | 62 | 1(2) | 62 |
|  | ORs | 10.33 | | 22.39 | | 1.29 | | 13.78 | | 7.75 | | 10.33 | |
|  |  | 3.88-27.50 | | 6.23-80.51 | | 0.14-12.15 | | 2.48-76.60 | | 2.01-29.90 | | 0.57-187.00 | |
| Fever | ＋ | 9 | 2 | 4(1) | 3 | 0 | 2 | 1(1) | 2 | 1(2) | 2 | 0(2) | 2 |
|  | － | 30 | 62 | 6 | 62 | 8 | 62 | 3 | 63 | 8 | 62 | 1 | 62 |
|  | ORs | 17.89 | | 13.78 | | － | | 10.33 | | 3.88 | | － | |
|  |  | 4.72-67.85 | | 2.48-76.60 | | － | | 0.72-148.54 | | 0.31-47.72 | | － | |
| Fatigue | ＋ | 17 | 4 | 6 | 4 | 1(1) | 4 | 3(1) | 4 | 5 | 4 | 1 | 4 |
|  | － | 30 | 62 | 6 | 62 | 8 | 62 | 3 | 62 | 8 | 62 | 1 | 62 |
|  | ORs | 8.78 | | 15.50 | | 1.94 | | 15.5 | | 9.69 | | 15.5 | |
|  |  | 2.72-28.39 | | 3.40-70.69 | | 0.19-19.55 | | 2.33-102.91 | | 2.15-43.72 | | 0.81-296.30 | |

irAEs; immune-related adverse events, ORs; odds ratios

Cases in parentheses are those with overlapping irAEs and are not included in the calculation of the odds ratio.

Supplemental Table4. Frequency of immune-related adverse events (irAEs) per signal symptom among the patients who were alive at least 6 months from the beginning of nivolumab (N=84)

|  | | irAEs | | Endocrine disorder | | Diarrhea, Colitis | | Pneumonitis | | Dermatitis | | Liver dysfunction | |
| --- | --- | --- | --- | --- | --- | --- | --- | --- | --- | --- | --- | --- | --- |
|  |  | ＋ | － | ＋ | － | ＋ | － | ＋ | － | ＋ | － | ＋ | － |
| Signal symptom | ＋ | 25 | 3 | 12(1) | 3 | 0 | 3 | 3(1) | 3 | 6(2) | 3 | 1(2) | 3 |
|  | － | 26 | 30 | 5 | 30 | 6(1) | 30 | 3(1) | 30 | 7(2) | 30 | 1(2) | 30 |
|  | ORs | 9.62 | | 24.00 | | － | | 10.00 | | 8.57 | | 3.33 | |
|  |  | 2.60-35.55 | | 4.94-116.54 | | － | | 1.36-73.33 | | 1.71-42.96 | | 0.26-42.93 | |
| Fever | ＋ | 9 | 1 | 4(1) | 1 | 0 | 1 | 1(1) | 1 | 1(2) | 1 | 0(2) | 1 |
|  | － | 26 | 30 | 5 | 30 | 6 | 30 | 3 | 30 | 7 | 30 | 1 | 30 |
|  | ORs | 10.39 | | 24.00 | | － | | 10.00 | | 4.29 | | － | |
|  |  | 1.23-87.53 | | 2.21-261.16 | | － | | 0.49-203.94 | | 0.24-77.22 | | － | |
| Fatigue | ＋ | 12 | 2 | 5 | 2 | 0 | 2 | 2 | 2 | 5 | 2 | 1 | 2 |
|  | － | 26 | 30 | 4 | 30 | 6 | 30 | 3 | 30 | 7 | 30 | 1 | 30 |
|  | ORs | 6.92 | | 12.00 | | － | | 10.00 | | 10.72 | | 15.00 | |
|  |  | 1.42-33.83 | | 1.72-83.81 | | － | | 1.01-98.88 | | 1.71-67.10 | | 0.66-339.57 | |

irAEs; immune-related adverse events, ORs; odds ratios

Cases in parentheses are those with overlapping irAEs and are not included in the calculation of the odds ratio.
